# Supplementary material for: Strand-specific single-cell methylomics reveals distinct modes of DNA demethylation dynamics during early mammalian development
Source: Nat Commun. 2021 Feb 24;12:1286. doi: 10.1038/s41467-021-21532-6 (PMC7904860; doi:10.1038/s41467-021-21532-6)
Supplement: Supplementary file 1 — Supplementary Information [file 41467_2021_21532_MOESM1_ESM.pdf]

Supplementary Information

Strand-specific single-cell methylomics reveals distinct modes of DNA demethylation dynamics during early mammalian development

Maya Sen[1†], Dylan Mooijman[1,7†], Alex Chialastri[2,3†], Jean-Charles Boisset[1], Mina Popovic[4], Björn Heindryckx[4], Susana M. Chuva de Sousa Lopes[4,5], Siddharth S. Dey[2,3,6]* & Alexander van Oudenaarden[1]*

[1] Oncode Institute, Hubrecht Institute-KNAW (Royal Netherlands Academy of Arts and Sciences) and University Medical Center Utrecht, Utrecht, The Netherlands.

[2] Department of Chemical Engineering, University of California Santa Barbara, Santa Barbara, CA 93106, USA.

[3] Center for Bioengineering, University of California Santa Barbara, Santa Barbara, CA 93106, USA.

[4] Ghent-Fertility and Stem cell Team (G-FaST), Department of Reproductive Medicine, Ghent University Hospital, 9000 Ghent, Belgium.

[5] Department of Anatomy and Embryology, Leiden University Medical Center, 2333 ZC Leiden, The Netherlands.

[6] Neuroscience Research Institute, University of California Santa Barbara, Santa Barbara, CA 93106, USA.

[7] Current address: Developmental Biology Unit, European Molecular Biology Laboratory, Heidelberg, Germany.

† These authors contributed equally to this work.

*Correspondence should be addressed to A.v.O. (a.vanoudenaarden@hubrecht.eu) and S.S.D (sdey@ucsb.edu).

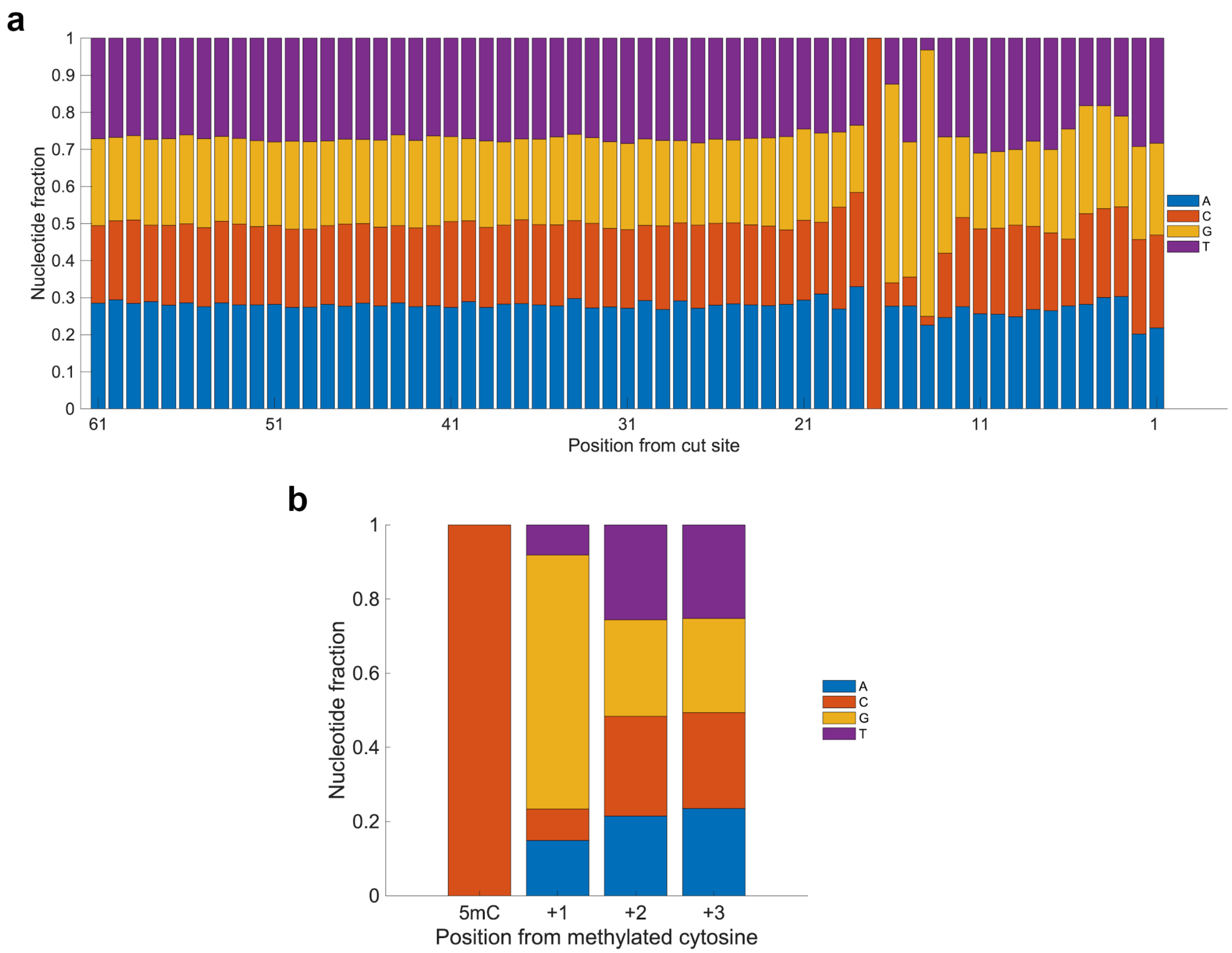

Supplementary Figure 1 | Nucleotide composition around methylated cytosine in scMspJI-seq and bisulfite sequencing. (**a**) Panel shows the nucleotide composition that was observed downstream of the MspJI cut site. In agreement with previous reports, MspJI was found to cut gDNA 16 bp downstream of the cut site[1]. In scMspJI-seq, an average of 44.0% (with a range of 31.0% to 59.9% in individual cells) of the methylated cytosines were found in a non-CpG context. (**b**) Analysis of nucleotide composition downstream of the methylated site in previously published single-cell whole-genome bisulfite sequencing[2]. In the bisulfite sequencing data, an average of 31.5% (with a range of 23.4% to 53.1% in individual cells) of the methylated cytosines were found in a non-CpG context. Both scMspJI-seq and bisulfite sequencing data are for E14 mESCs.

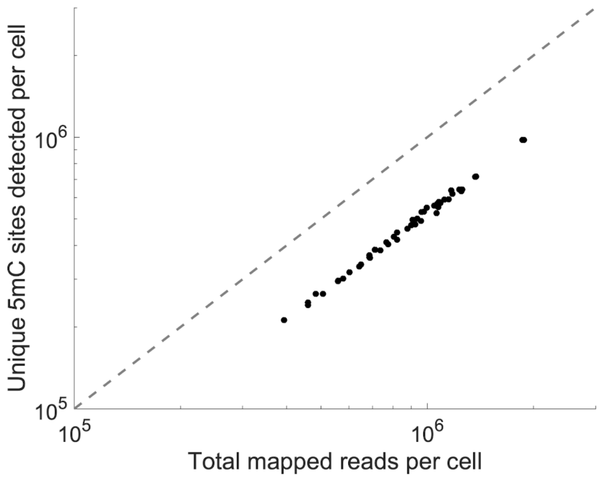

Supplementary Figure 2 | Number of unique 5mC sites detected in scMspJI-seq. The figure shows the number of unique 5mC sites detected per cell as a function of the sequencing depth. The number of unique 5mC sites detected per cell ranged from 212,000 to 977,000, with a median of 484,000 5mC sites per cell. The number of unique 5mC sites detected per cell is increasing monotonically with the sequencing depth, suggesting that more unique sites could be detected per cell by sequencing the Illumina libraries deeper. The figure shows data from E14 mESCs.

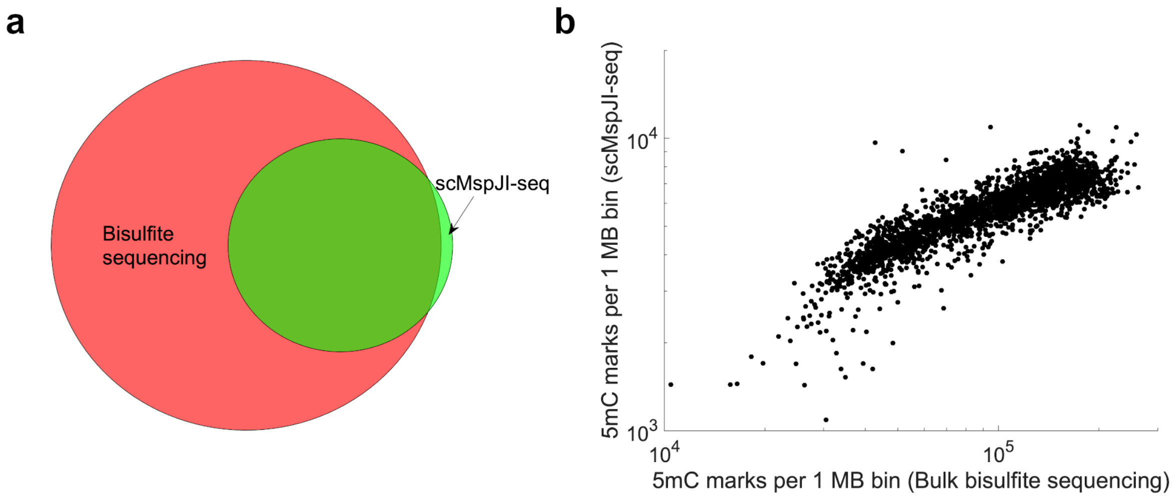

Supplementary Figure 3 | Comparison of scMspJI-seq to bulk bisulfite sequencing. (**a**) Venn diagram shows that 97.2% of the 5mCpG sites detected in single E14 cells by scMspJI-seq is also found in bulk bisulfite sequencing of E14 gDNA. (**b**) Number of DNA methylation marks detected within 1 MB bins in scMspJI-seq correlates well with the bulk bisulfite sequencing methylome (Pearson *r* = 0.84).

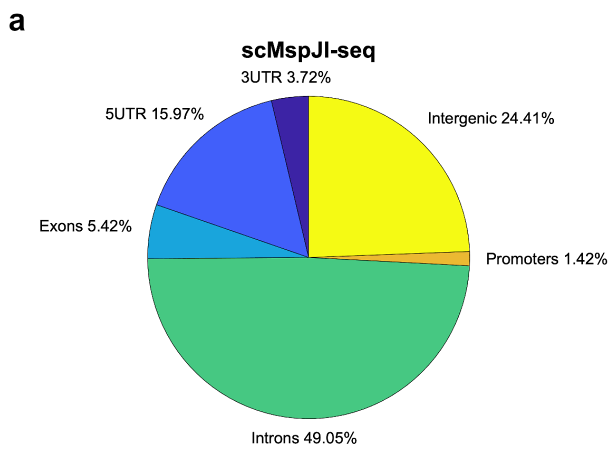

Supplementary Figure 4 | Distribution of 5mC over different genomic elements. (**a**) Pie chart showing the distribution of 5mC sites over promoters, 5' UTRs, exons, introns, 3' UTRs, and intergenic regions in scMspJI-seq. (**b**) Pie chart showing the distribution of 5mC sites over the same genomic elements in whole-genome bisulfite sequencing[2]. The figure shows data from E14 mESCs.

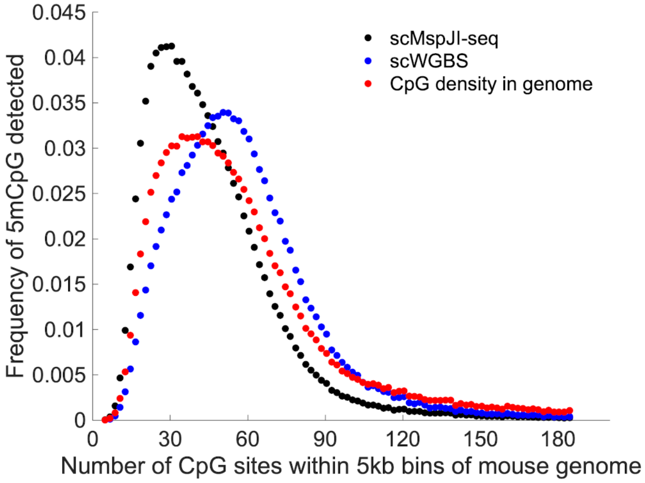

Supplementary Figure 5 | Distribution of 5mCpG sites over genomic regions of varying CpG density. The red curve show the distribution of CpG sites over 5 kb bins of the mouse genome. The black and blue curves show the distribution of 5mCpG sites that are detected in genomic bins of different CpG densities in scMspJI-seq and scWGBS, respectively[2]. scMspJI-seq is slightly biased towards the detection of 5mCpG within genomic regions that have lower CpG density. The figure shows data from E14 mESCs.

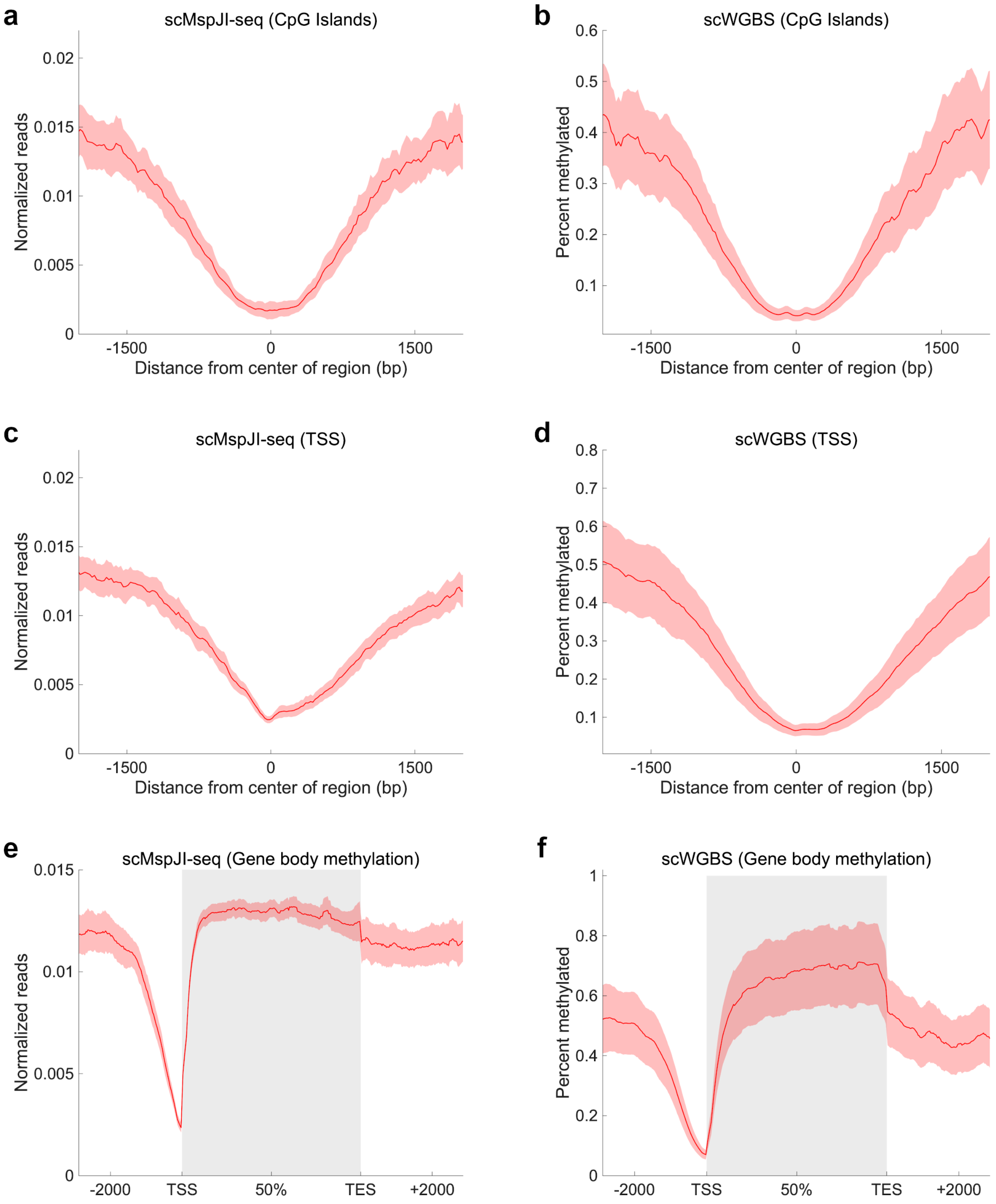

Supplementary Figure 6 | Genome-wide DNA methylation landscapes. (**a**,**b**) Panels showing hypomethylation at CpG islands in scMspJI-seq and scWGBS, respectively[2]. (**c**,**d**) Panels showing hypomethylation at transcription start sites (TSS) in scMspJI-seq and scWGBS, respectively. (**e**,**f**) Gene body DNA methylation profiles obtained from scMspJI-seq and scWGBS,

respectively. Shaded red regions indicate standard deviations in the distribution of 5mC. The figure shows data from E14 mESCs.

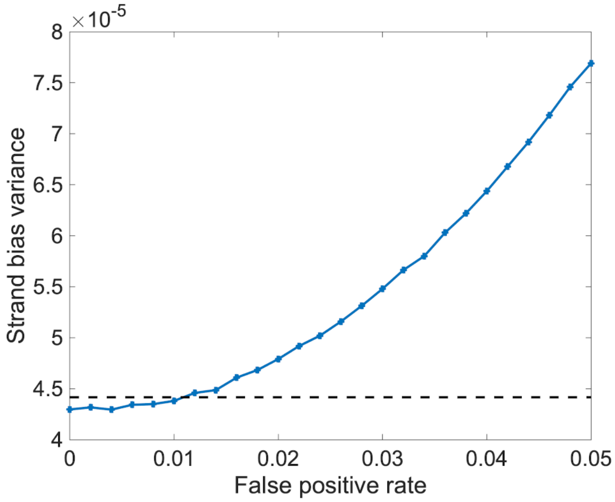

Supplementary Figure 7 | False positive detection rate of 5hmC in scMspJI-seq. The panel shows that the variance of the simulated strand bias distribution increases with higher rates of 5hmC false positive detection in scMspJI-seq (blue line). The dashed black line indicates the experimental strand bias variance obtained from applying scMspJI-seq to E14 mESCs. Data from scMspJI-seq and scAba-seq were combined to quantify the false positive detection rate of 5hmC in scMspJI-seq[3]. For different efficiencies of 5mC *vs.* 5hmC detection, a mathematical model was built where 5mC and 5hmC sites were drawn from a binomial distribution and distributed on the two DNA strands of a chromosome using the strand bias distributions from scMspJI-seq and scAba-seq[3]. By comparing the variance of the experimental strand bias distribution to that obtained from the simulations, the false-positive detection rate of 5hmC was estimated to be around 1.1%.

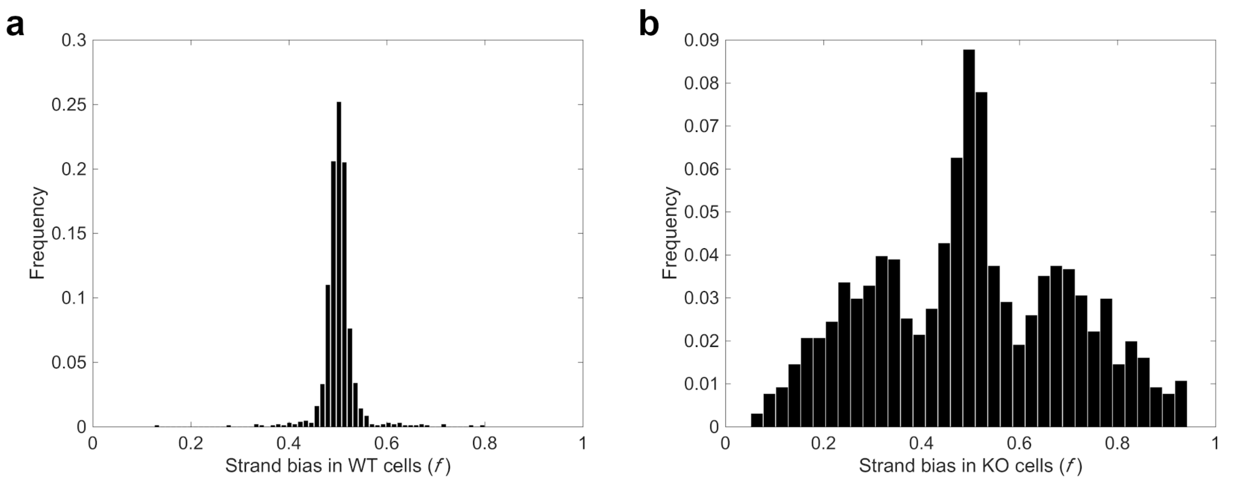

Supplementary Figure 8 | Strand-specific detection of 5mC in single cells using scMspJI-seq. (**a**) Chromosomes in E14 cells show a tight strand bias distribution centered around 0.5. (**b**) CRISPR-Cas9 mediated knockout of *Dnmt1* in E14 cells results in a dramatic increase in the width of the strand bias distribution indicating loss of maintenance methylation and the ability of scMspJI-seq to quantify strand-specific 5mC in single cells.

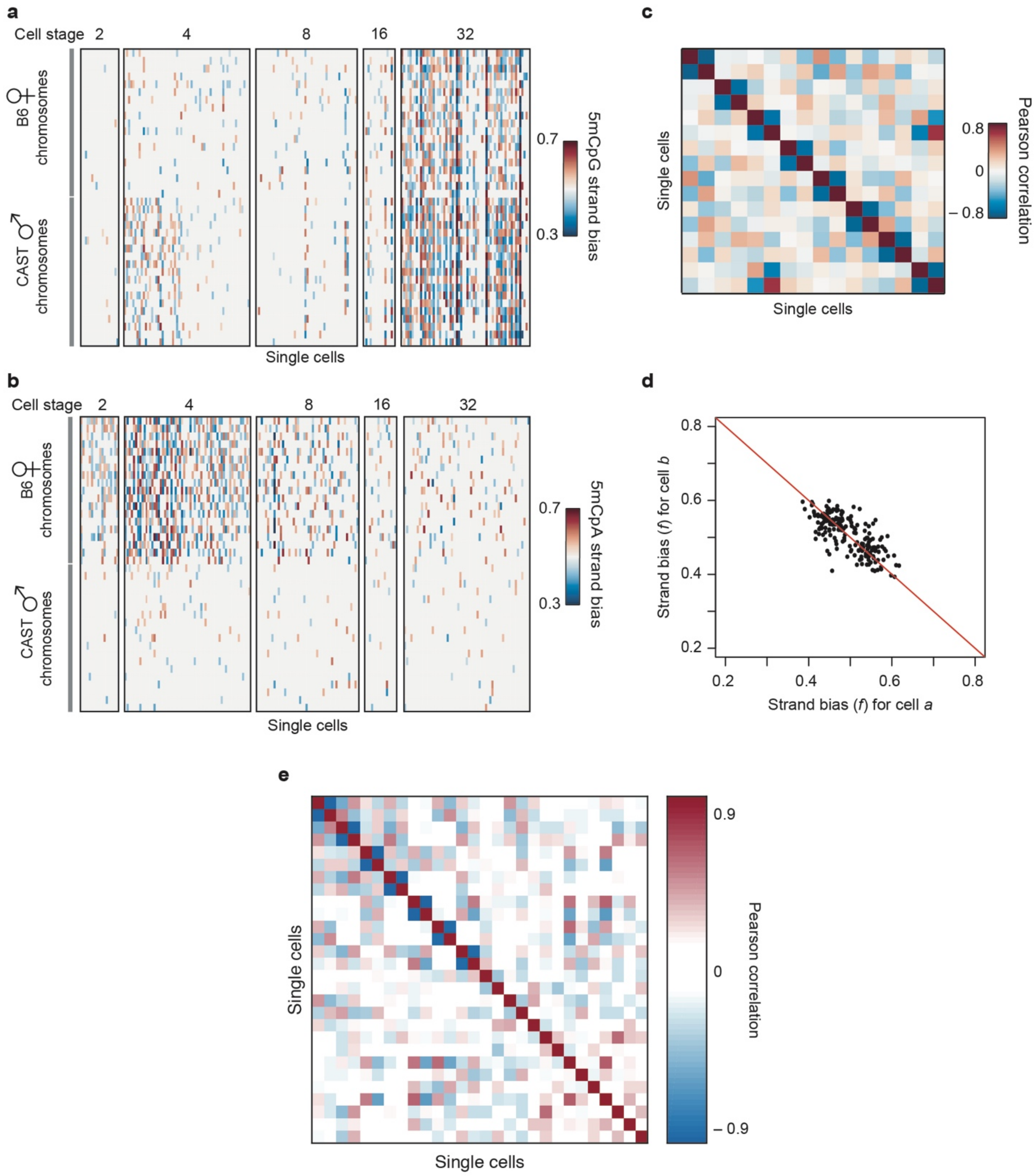

Supplementary Figure 9 | DNA demethylation dynamics in preimplantation mouse embryos. (**a**) Heatmap shows 5mCpG strand bias for all maternal and paternal chromosomes from the 2- to 32-cell stage of development. The data shows a dramatic increase in 5mCpG strand bias from the 16- to 32-cell stage of development. (**b**) Heatmap shows 5mCpA strand bias for all maternal and paternal chromosomes from the 2- to 32-cell stage of development. For a majority

of cells at the 2- and 4-cell stage, the maternal genome displays 5mCpA strand bias that deviates from 0.5. (**c**) Heatmap shows Pearson correlation for the maternal 5mCpA strand bias between pairs of cells at the 2-cell stage of development. (**d**) Pairs of cells in **c** that display strongly anticorrelated 5mCpA strand bias are shown here, suggesting that we can use this method to identify sister cells at the 2-cell stage of development. (**e**) Heatmap shows Pearson correlation for the paternal 5mCpG strand bias between pairs of cells (within the bimodal strand bias distribution) at the 32-cell stage of development. Strongly negative Pearson correlations indicate that we can identify sister cells within 32-cell stage embryos.

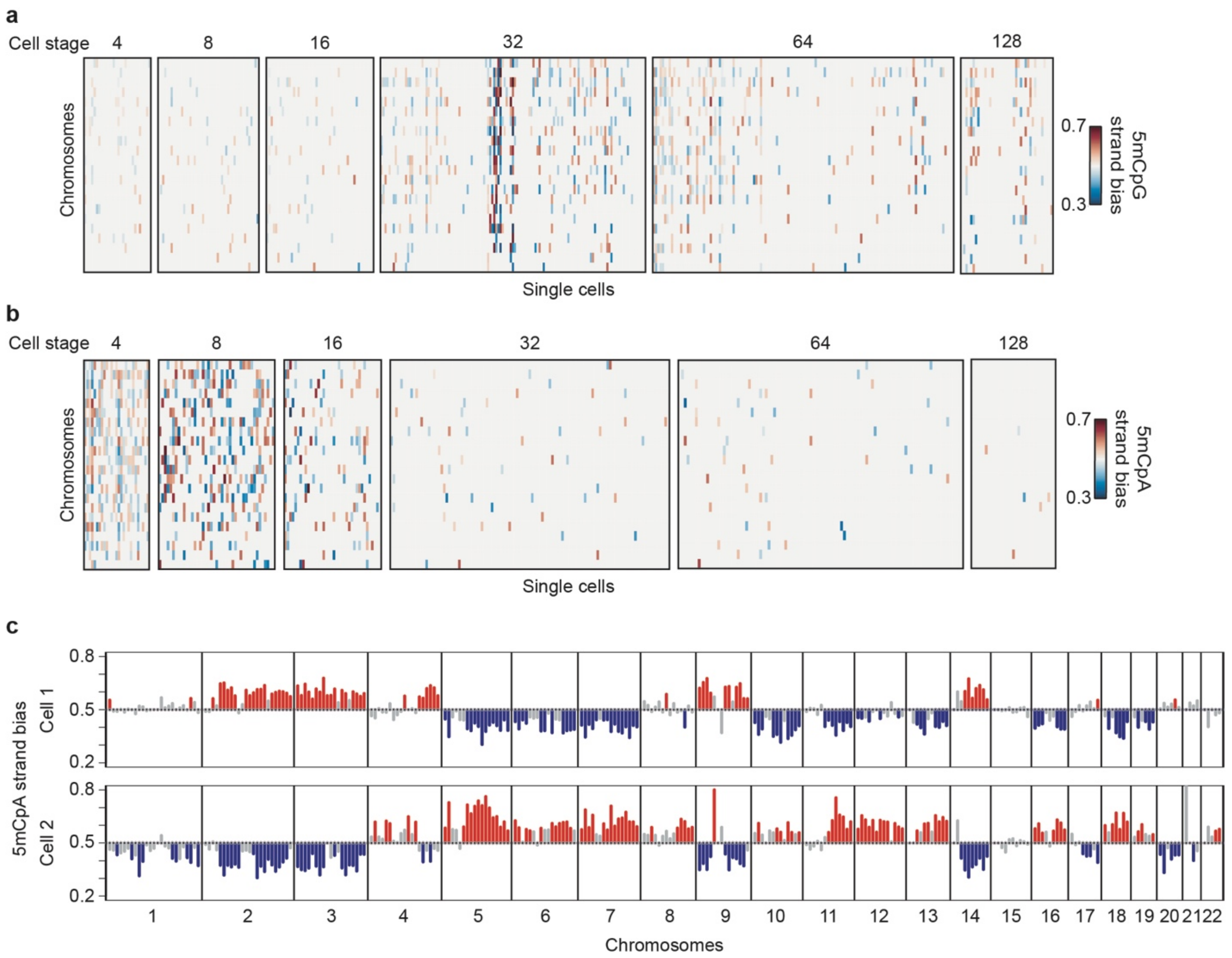

Supplementary Figure 10 | DNA demethylation dynamics in preimplantation human embryos. (**a**) Heatmap shows 5mCpG strand bias for all chromosomes from the 4- to 128-cell stage of human development. (**b**) Heatmap shows 5mCpA strand bias for all chromosomes from the 4- to 128-cell stage of human development. 5mCpA strand bias deviates from 0.5 for a large number of chromosomes till the 16-cell stage. (**c**) An example of a pair of cells that display strongly anti-correlated 5mCpA strand bias along the entire genome.

Supplementary Table 1 | Table listing 8 bp cell-specific barcodes used in scMspJI-seq

Cell barcode	Sequence
1	GCGAGATT
2	CATTCCAC
3	CCGATGAT
4	AGCTTAGC
5	CGTTACTG
6	TTCGCTTG
7	CTACTGCT
8	AAGAGAGC
9	AACTGTGG
10	CACATCAG
11	AGTCAGTC
12	CGTTGTCA
13	TAGGAACG
14	CCTGATCT
15	AACGAGCA
16	GCAGTAAC
17	TTCTCGAC
18	GTCCAATC
19	AACTCACC
20	CTGCGAAT
21	ACGTTACC
22	AGTTGCAC
23	AATAGCCG
24	ACCTCTAC
25	TTCGACGT
26	TTGATCCG
27	GTACAGGT
28	ACCACCTT
29	GGATTCGA
30	CCGTTAAG
31	GTTCGGAA
32	CCACCATT
33	CGATCGAT
34	GTCTGTAC
35	ACGCCTTA

36	CAGGATTC
37	GGAAGATC
38	TCAGACGA
39	TGCGCTAA
40	GAGAATGC
41	TTAGCGTG
42	TGAAGGCT
43	CTTAGCAG
44	AAGCTACC
45	ACATCTGC
46	CGCATTAC
47	CCTAGATC
48	CATCCAGA
49	GGTCTTGA
50	GACAGATG
51	GAACAGCT
52	ACGAGCAA
53	TCCTTCTC
54	GCGTGTAA
55	CAGCCATA
56	GAATTGCC
57	AATCAGCC
58	CTCAACAC
59	GCAGATAC
60	TCGCTTGT
61	AGTCTTCG
62	TAGAGGCA
63	CCTTGGTT
64	AGAACGCA
65	GTATACGC
66	ACTGCTAG
67	ATCGGTGA
68	GACCATGA
69	TCCAAGGT
70	GCCAACAT
71	GCGTCAAT
72	AGCCAAGT
73	ACGTCAGA
74	TCACCTGA

75	GCAATCCT
76	AATTCGCC
77	TGAAGCTC
78	GTCCGATA
79	CCTGTAGT
80	CAGACTGT
81	TGTAGCCT
82	GATGCCAT
83	AACGGCAT
84	GATAGCAC
85	TACGGTTC
86	TGGTTGGA
87	TCGTGTAC
88	TAGCGGAA
89	CTAGGCTA
90	GCTGTGTA
91	CAGGTCTT
92	AAGAGCCA
93	GCATGACT
94	TTACGGTC
95	ACGCATAC
96	GATGCAAC

Supplementary References

1. Cohen-Karni, D. *et al.* The MspJI family of modification-dependent restriction endonucleases for epigenetic studies. *Proc. Natl. Acad. Sci. U.S.A.* **108,** 11040–11045 (2011).
2. Smallwood, S. A. *et al.* Single-cell genome-wide bisulfite sequencing for assessing epigenetic heterogeneity. *Nat. Methods* **11,** 817–820 (2014).
3. Mooijman, D., Dey, S. S., Boisset, J. C., Crosetto, N. & van Oudenaarden, A. Single-cell 5hmC sequencing reveals chromosome-wide cell-to-cell variability and enables lineage reconstruction. *Nat. Biotechnol.* **34,** 852–856 (2016).
