# Supplementary material for: Strand-specific single-cell methylomics reveals distinct modes of DNA demethylation dynamics during early mammalian development
Source: Nat Commun. 2021 Feb 24;12:1286. doi: 10.1038/s41467-021-21532-6 (PMC7904860; doi:10.1038/s41467-021-21532-6)
Supplement: Supplementary file 3 — Description of Additional Supplementary Files [file 41467_2021_21532_MOESM3_ESM.pdf]

Description of Additional Supplementary Files

Supplementary Software: The zipped file “process_scmspji.zip” contains a script, a readme text file with all information to run the script, and a list of barcodes.
