# Supplementary material for: Strand-specific single-cell methylomics reveals distinct modes of DNA demethylation dynamics during early mammalian development
Source: Nat Commun. 2021 Feb 24;12:1286. doi: 10.1038/s41467-021-21532-6 (PMC7904860; doi:10.1038/s41467-021-21532-6)
Supplement: Supplementary file 4 — Supplementary Software [file 41467_2021_21532_MOESM4_ESM.zip › Readme.rtf]

The custom code to demultiplex the data, identify 5mC genomic position, strand information, and remove PCR duplicates was written in Perl.The zipped folder contains the Perl script (process_scmspji.pl) and the barcode file (mspj1.txt).To run the Perl script (process_scmspji.pl), use the following syntax: process_scaba.pl genome_file.fa sam_file.sam msjp1.txtHere, the first field corresponds to the genome file, the second field corresponds to the sam file produced from a BWA aligner and the third field corresponds to the barcode file. The output contains two important files:(1) *.faba: This contains the final data file with the following information that is used in the manuscriptColumn 1: Cell numberColumn 2: ChromosomeColumn 3: CoordinateColumn 4: Strand information (+1: Plus strand, -1: Minus strand)Column 5: UMIColumn 6: The first base is the methylated cytosine and the next three bases is the downstream genomic sequence(2) *.raba: This contains the same information as *.faba but without the PCR and other duplicates removed.The Perl script can be run on the raw data provided in GEO: GSE139984. The output should correspond to the processed data submitted in GEO: GSE139984. The expected run time depends on the size of the sam file.
