# Supplementary material for: Strand-specific single-cell methylomics reveals distinct modes of DNA demethylation dynamics during early mammalian development
Source: Nat Commun. 2021 Feb 24;12:1286. doi: 10.1038/s41467-021-21532-6 (PMC7904860; doi:10.1038/s41467-021-21532-6)
Supplement: Supplementary file 5 — Reporting summary [file 41467_2021_21532_MOESM5_ESM.pdf]

nature research

Corresponding author(s): Siddharth Dey and Alexander van Oudenaarden

Last updated by author(s): Dec 21, 2020

Reporting Summary

Nature Research wishes to improve the reproducibility of the work that we publish. This form provides structure for consistency and transparency in reporting. For further information on Nature Research policies, see Authors & Referees and the Editorial Policy Checklist .

Statistics

For all statistical analyses, confirm that the following items are present in the figure legend, table legend, main text, or Methods section.

n/a	Confirmed	
☐	☒	The exact sample size (*n*) for each experimental group/condition, given as a discrete number and unit of measurement
☐	☒	A statement on whether measurements were taken from distinct samples or whether the same sample was measured repeatedly
☐	☒	The statistical test(s) used AND whether they are one- or two-sided<br>*Only common tests should be described solely by name; describe more complex techniques in the Methods section.*
☒	☐	A description of all covariates tested
☒	☐	A description of any assumptions or corrections, such as tests of normality and adjustment for multiple comparisons
☐	☒	A full description of the statistical parameters including central tendency (e.g. means) or other basic estimates (e.g. regression coefficient) AND variation (e.g. standard deviation) or associated estimates of uncertainty (e.g. confidence intervals)
☐	☒	For null hypothesis testing, the test statistic (e.g. *F*, *t*, *r*) with confidence intervals, effect sizes, degrees of freedom and *P* value noted<br>*Give P values as exact values whenever suitable.*
☒	☐	For Bayesian analysis, information on the choice of priors and Markov chain Monte Carlo settings
☒	☐	For hierarchical and complex designs, identification of the appropriate level for tests and full reporting of outcomes
☐	☒	Estimates of effect sizes (e.g. Cohen's *d*, Pearson's *r*), indicating how they were calculated

Our web collection on statistics for biologists contains articles on many of the points above.

Software and code

Policy information about availability of computer code

Data collection	Next-generation sequencing libraries were sequenced on an Illumina NextSeq 500 platform.
Data analysis	Sequencing reads containing the correct cell-specific barcode were mapped to the mouse (mm10) or human (hg19) genome using the Burrows-Wheeler Aligner (BWA, version 0.7.15) and filtered for uniquely mapping reads to the genome. A custom script was written in Perl to demultiplex the sam files, identify 5mC position, strand information, and remove PCR duplicates. The custom script is included as a zipped Supplementary File.

For manuscripts utilizing custom algorithms or software that are central to the research but not yet described in published literature, software must be made available to editors/reviewers. We strongly encourage code deposition in a community repository (e.g. GitHub). See the Nature Research guidelines for submitting code & software for further information.

Data

Policy information about availability of data

All manuscripts must include a data availability statement. This statement should provide the following information, where applicable:

- Accession codes, unique identifiers, or web links for publicly available datasets
- A list of figures that have associated raw data
- A description of any restrictions on data availability

Data can be openly accessed at Gene Expression Omnibus (GEO). Accession Code GEO: GSE139984. (https://www.ncbi.nlm.nih.gov/geo/query/acc.cgi?acc=GSE139984)
Figures associated with raw data. Figure 2a-e; Figure 3a-d; Figure 4a-f; Figure 5a,b; Supplementary Figure 1a,b; Supplementary Figure 2; Supplementary Figure 3a,b; Supplementary Figure 4a,b; Supplementary Figure 5; Supplementary Figure 6a-f; Supplementary Figure 7; Supplementary Figure 8a,b; Supplementary Figure 9a-e; Supplementary Figure 10a-c.
There are no restrictions on data availability.

Field-specific reporting

Please select the one below that is the best fit for your research. If you are not sure, read the appropriate sections before making your selection.

☒ Life sciences ☐ Behavioural & social sciences ☐ Ecological, evolutionary & environmental sciences

For a reference copy of the document with all sections, see nature.com/documents/nr-reporting-summary-flat.pdf

Life sciences study design

All studies must disclose on these points even when the disclosure is negative.

Sample size	No statistical calculations were performed to estimate sample size. Sample sizes were chosen to robustly estimate the quantities described in the main manuscript
Data exclusions	All data was included except data from single cells that failed to sequence. This includes experiments described in Figures 2, 4 and 5.
Replication	Multiple embryos were sequenced from each stage of development. Details of embryos and replicates are provided in the legend of Figure 4. All replication attempts were successful.
Randomization	Single embryonic stem cells were randomly obtained by FACS sorting. Single cells from embryos were randomly obtained by manual pipetting. No other randomization was performed.
Blinding	Blinding was not relevant to this study as no manual assessment of experiments was performed. All data obtained from experiments were assessed in the same way using the analysis pipelines and scripts described above and in the main manuscript.

Reporting for specific materials, systems and methods

We require information from authors about some types of materials, experimental systems and methods used in many studies. Here, indicate whether each material, system or method listed is relevant to your study. If you are not sure if a list item applies to your research, read the appropriate section before selecting a response.

Materials & experimental systems

n/a	Involved in the study	
☒	☐	Antibodies
☐	☒	Eukaryotic cell lines
☒	☐	Palaeontology
☐	☒	Animals and other organisms
☐	☒	Human research participants
☒	☐	Clinical data

Methods

n/a	Involved in the study	
☒	☐	ChIP-seq
☒	☐	Flow cytometry
☒	☐	MRI-based neuroimaging

Eukaryotic cell lines

Policy information about cell lines

Cell line source(s)	E14tg2a mouse embryonic stem cells were obtained from American Type Culture Collection (ATCC CRL-182) and the hybrid 129/Sv:CAST/EiJ mouse embryonic stem cells were obtained from Jop Kind's group (Hubrecht Institute).
Authentication	The cell lines were not authenticated
Mycoplasma contamination	Cell lines were tested negative for mycoplasma.
Commonly misidentified lines (See ICLAC register)	No commonly misidentified cell lines were used in the study.

Animals and other organisms

Policy information about studies involving animals; ARRIVE guidelines recommended for reporting animal research

Laboratory animals	CAST/EiJ male and C57BL/6 female mice were used in this study. CAST/EiJ x C57BL/6 hybrid mouse embryos were obtained from four 3-month-old superovulated C57BL/6 mothers. The male CAST/EiJ mice used in this study were 21-35 days old.
Wild animals	This study did not involve wild animals.

Field-collected samples	This study did not involve samples collected from the field.
Ethics oversight	All animal experiments were approved by the Royal Netherlands Academy of Arts and Sciences and were performed according to the animal experimentation guidelines of the KNAW.

Note that full information on the approval of the study protocol must also be provided in the manuscript.

Human research participants

Policy information about studies involving human research participants

Population characteristics	Supernumerary cryopreserved human embryos were obtained for research from patients undergoing in vitro fertilization (IVF) using standard clinical protocols, at the Department for Reproductive Medicine, Ghent University Hospital. Embryos were obtained from 16 patients with a mean age of 33 (Median = 33, Standard deviation = 3.76).
Recruitment	All embryos were donated following patients' written informed consent.
Ethics oversight	This study was approved by the Ghent University Institutional Review Board (EC2015/1114) and the Belgian Federal Commission for medical and scientific research on embryos in vitro (ADV_060_UZGent).

Note that full information on the approval of the study protocol must also be provided in the manuscript.
